# A Treatise on Plague

**Published:** 1902-09

**Authors:** 


**A Treatise on Plague.** By Major GEORGE S. THOMSON and Dr. JOHN THOMSON. Pp. xvi., 299. London: Swan Sonnenschein and Co., Lim. 1901.

**La Peste Bubonique dans la République Argentine et au Paraguay Épidémies de 1899—1900.** Rapport présenté au Département National d'Hygiène par les Docteurs LUIS AGOTE et A. J. MEDINA. Pp. xxv., 299. Buenos Aires: Félix Lajouane. 1901.

The first of these books contains a quantity of valuable information about plague as it has appeared in India. The authors contend that the chief conditions favouring "precipitation and propagation" of plague are, in order of importance, (1) want of fresh air, (2) overcrowding, (3) filth. They consider that "the only reliable measure is the demolition of insanitary dwellings in the crowded and filthy quarters of our towns and their reconstruction on improved sanitary principles, and a system of skilled supervision by a reliable staff of sanitary officers and subordinates." They do not hesitate to denounce other methods which have been employed or recommended in unsparing terms. Thus they say: "First it was the use of the whitewash brush, now the abuse of the rat. Imbecility could hardly go further or show greater impotency," &c. No doubt improved sanitation still requires energetic advocacy, and nowhere more than in the crowded poor quarters of Indian cities; but we do not think that any scientific cause is advanced by polemics of this kind. The authors would have done better to confine themselves to bringing forward conclusive evidence that their view that want of free supply of pure air is *the* factor in the spread of plague. They give an account of plague prophylactic inoculation, and say with regard to it: "We are convinced of the protective power of Professor Haffkine's prophylactic inoculation. We are of opinion that of all the temporary measures for combating the plague known up to now inoculation is the most reliable and safe measure." But they add that it can never be the sole remedy against plague, and by leading to a sense of false security it may interfere with the abolition of the disease by postponing the carrying out of the more drastic and permanently efficacious measures. An account is given of an epidemic in Satara City, and a valuable analysis of the reports of the Parel Hospital. The book opens with an historical retrospect of the disease which is marred by the introduction of irrevelant matter. Indeed, the whole work suffers from undue redundancy, discursiveness, and want of arrangement, which makes it somewhat difficult reading. Those however who can get over these faults and also the polemical manner in which the authors urge their views against the opinions of others, will find that the book contains much matter of interest.

The second work is an interesting and well-written account of the plague in the Argentine Republic and in Paraguay. The clinical descriptions are clear, and help one greatly in forming a mental picture of cases of the disease. In Paraguay, as in other epidemics, the progress of the disease was at first slow and the early stages benign. In the outbreak at Rosario we notice the early and probably inevitable mistakes in diagnosis, and the reluctance to admit the existence of plague in the town. In all these points we are reminded of the story of the Great Plague in London as given us by Defoe. When the disease reached Buenos Ayres the authorities there profited by the errors of the other towns, and prompt measures were taken against it with great success, and to this happy result the good sanitary state of the city seems to have largely contributed. The authors bring forward evidence to show that recrudescences of plague occur when the two conditions of great dryness of the atmosphere and a moderate temperature concur. Both cold and excessive heat are inimical to the spread of plague. Their observations emphasize the importance of the rat in spreading the disease. An appendix gives full reports of the clinical symptoms and *post-mortem* findings in ninety cases. The book will well repay perusal.

